
JOURNAL INFORMATION
==============================
NLM Title Abbreviation: EURASIP J Bioinform Syst Biol
Journal ID: EURASIP J Bioinform Syst Biol
NLM Journal ID: 101263720

EURASIP Journal on Bioinformatics and Systems Biology

ISSN: 1687-4145
EISSN: 1687-4153
Publisher: Springer

ARTICLE INFORMATION
==============================
PMCID: PMC3171352
PMID: 18301723
DOI: 10.1155/2007/79128
Article ID: 1687-4153-2007-79128
Article version: 1
Subjects: Editorial

Information Theoretic Methods for Bioinformatics

Rissanen Jorma 1 2 jrrissanen@yahoo.com
Grünwald Peter 3 peter.grunwald@cwi.nl
Heikkonen Jukka 4 jukka.heikkonen@tkk.fi
Myllymäki Petri 2 5 petri.myllymaki@cs.helsinki.fi
Roos Teemu 2 5 teemu.roos@hiit.fi
Rousu Juho 5 juho.rousu@cs.helsinki.fi
1 Computer Learning Research Center, University of London, Royal Holloway, TW20 0EX, UK
2 Helsinki Institute for Information Technology, University of Helsinki, P.O. Box 68, Helsinki 00014, Finland
3 Centrum voor Wiskunde en Informatica (CWI), P.O. Box 94079, Amsterdam 1090 GB, The Netherlands
4 Laboratory of Computational Engineering, Helsinki University of Technology, P.O. Box 9203, 02015 HUT, Finland
5 Department of Computer Science, University of Helsinki, P.O. Box 68, Helsinki 00014, Finland
Publication date: 2007
Electronic publication date: 2008 Feb 13
Volume: 2007
Issue: 1
First page: 79128
Last page: 79128
Received 2007 Dec 24; Accepted 2007 Dec 24
Copyright: Copyright © 2007 Jorma Rissanen et al.
Copyright year: 2007
Copyright holder: Jorma Rissanen et al.
License: This is an open access article distributed under the Creative Commons Attribution License, which permits unrestricted use, distribution, and reproduction in any medium, provided the original work is properly cited.
License URL: https://creativecommons.org/licenses/by/4.0/

==============================

Hindawi Publishing Corporation
EURASIP Journal on Bioinformatics and Systems Biology
Volume 2007, Article ID 79128, 2 pages
doi:10.1155/2007/79128

*Editorial*

### Information Theoretic Methods for Bioinformatics

**Jorma Rissanen,[1,2] Peter Grünwald,[3] Jukka Heikkonen,[4] Petri Myllymäki,[2,5] Teemu Roos,[2,5] and Juho Rousu[5]**

[1] *Computer Learning Research Center, University of London, Royal Holloway TW20 0EX, UK*
[2] *Helsinki Institute for Information Technology, University of Helsinki, P.O. Box 68, 00014 Helsinki, Finland*
[3] *Centrum voor Wiskunde en Informatica (CWI), P.O. Box 94079, 1090 GB Amsterdam, The Netherlands*
[4] *Laboratory of Computational Engineering, Helsinki University of Technology, P.O. Box 9203, 02015 HUT, Finland*
[5] *Department of Computer Science, University of Helsinki, P.O. Box 68, 00014 Helsinki, Finland*

Received 24 December 2007; Accepted 24 December 2007

Copyright © 2007 Jorma Rissanen et al. This is an open access article distributed under the Creative Commons Attribution License, which permits unrestricted use, distribution, and reproduction in any medium, provided the original work is properly cited.

The ever-ongoing growth in the amount of biological data, the development of genome-wide measurement technologies, and the gradual, inevitable shift in molecular biology from the study of individual genes to the systems view; all these factors contribute to the need to study biological systems by statistical and computational means. In this task, we are facing a dual challenge: on the one hand, biological systems and hence their models are inherently complex, and on the other hand, the measurement data, while being genome-wide, are typically scarce in terms of sample sizes (the "large $p$, small $n$" problem) and noisy.

This means that the traditional statistical approach, where the model is viewed as a distorted image of something called a true distribution which the statisticians are trying to estimate, is poorly justified. This lack of rationality is particularly striking when one tries to learn the structure of the data by testing for the truth of a hypothesis in a collection where none of them is true. Similarly, the Bayesian approaches that require prior knowledge, which is either nonexistent or vague and difficult to express in terms of a distribution for the parameters, are subject to modeling assumptions which may bias the results in an unintended manner.

It was the editors' intent and hope to encourage applications of techniques for model fitting influenced by information theory, originally created for communication theory but more recently expanded to cover algorithmic information theory and applicable to statistical modeling. In this view, the objective in modeling is to learn structures and properties in data by simply fitting models without requiring any of them to be "true". The performance is not measured by any distance to the nonexisting "truth" but in terms of the probability they assign to the data, which is equivalent to the code length with which the data can be encoded, taking advantage of the regular features the model prescribes to the data. This task requires information and coding theoretic means. Similarly, the frequently used distance measures like the Kullback-Leibler divergence and the mutual information express mean codelength differences.

D. Benedetto et al. study correlations and compressibility of proteome sequences. They identify dependencies at the range of 10 to 100 amino acids. The source of such dependencies is not entirely clear. One contributing factor in the case of interprotein dependencies is likely to be sequence duplication. The dependencies can be exploited in compression of proteome sequences. Furthermore, they seem to have a role in evolutionary and structural analysis of proteomes.

C. M. Hemmerich and S. Kim also use information theory for studying the correlations in protein sequences. They base their method on computing the mutual information of nonadjacent residues lying at a fixed distance $d$ apart, where the distance is varied from zero to a fixed upper bound. The mutual information vector formed by these statistics is used to train a nearest-neighbor classifier to predict membership in protein families with results indicating that the correlations between nonadjacent residues are predictive of protein family.

H. M. Aktulga et al. detect statistically dependent genomic sequences. Their paper addresses two applications. First, they identify different parts of a gene (maize zmSRp32) that are mutually dependent without appealing to the usual assumption that dependencies are revealed by a considerable amount of exact matches. It is discovered that dependencies exist between the 5′ untranslated region and its alternatively spliced exons. As a second application, they discover short tandem repeats which are useful in, for instance, genetic profiling. In both cases, the used techniques are based on mutual information.

The objective in the paper by A. Rao et al. is to discover long-range regulatory elements (LREs) that determine tissue-specific gene expression. Their methodology is based on the concept of *directed information*, a variant of mutual information introduced originally in the 1970s. It is shown that directed information can be successfully used for selecting motifs that discriminate between tissue-specific and nonspecific LREs. In particular, the performance of directed information is better than that of mutual information.

F. Fabris et al. present an in-depth study to BLOSUM—block substitution matrix scores. They propose a decomposition of the BLOSUM score into three components: the mutual information of two compared sequences, the divergence of observed amino acid co-occurence frequencies from the probabilities in the substitution matrix, and the background frequency divergence measuring the stochastic distance of the observed amino acid frequences from the marginals in the substitution matrix. The authors show how the result of the decomposition, called BLOSpectrum, can be used to analyze questions about the correctness of the chosen BLOSUM matrix, the degree of typicality of compared sequences or their alignment, and the presence of weak or concealed correlations in alignments with low BLOSUM scores.

The paper by J. Conery presents a new framework for biological sequence alignment that is based on describing pairs of sequences by simple regular expressions. These regular expressions are given in terms of right-linear grammars, and the best grammar is found by use of the MDL principle. Essentially, when two sequences contain similar substrings, this similarity can be exploited to describe the sequences with fewer bits. The precise codelengths are determined with a substitution matrix that provides conditional probabilities for the event that a particular symbol is replaced by another particular symbol. One advantage of such a grammar-based approach is that gaps are not needed to align sequences of varying length. The author experimentally compares the alignments found by his method with those found by CLUSTALW. In a second experiment, he measures the accuracy of his method on pairwise alignments taken from the BAlisBASE benchmark.

S. C. Evans et al. explore miRNA sequences based on MDLcompress, an MDL-based grammar inference algorithm that is an extension of the optimal symbol compression ratio (OSCR) algorithm published earlier. Using MDLcompress, they analyze the relationship between miRNAs, single nucleotide polymorphisms (SNPs) and breast cancer. Their results suggest that MDLcompress outperforms other grammar-based coding methods, such as DNA sequitur, while retaining a two-part code that highlights biologically significant phrases. The ability to quantify cost in bits for phrases in the MDL model allows prediction of regions where SNPs may have the most impact on biological activity.

The partially redundant third position of codons (protein-coding nucleotide triplets) tends to have a strongly biased distribution. The amount of bias is known to be correlated with G+C (guanine-cytosine) composition in the genome. In their paper, H. Suzuki et al. quantify the correlation of G+C composition with synonymous codon usage bias, where the bias is measured by the entropy of the third codon position. They show that the correlation depends on various genomic features and varies among different species. This raises several interesting questions about the different evolutionary forces causing the codon usage bias.

The paper by P. E. Meyer et al. tackles the challenging problem of inferring large gene regulatory networks using information theory. Their MRNET method extends the maximum relevance/minimum redundancy (MRMR) feature selection technique to networks by formulating the network inference problem as a series of input/output supervised gene selection procedures. Empirical results are competitive with the state-of-the-art methods.

P. Kontkanen et al. study the problem of computing the normalized maximum likelihood (NML) universal model for Bayesian networks, which are important tools for modeling discrete data in biological applications. The most advanced MDL method for model selection between such networks is based on comparing the NML distributions for each network under consideration, but the naive computation of these distributions requires exponential time with respect to the given data sample size. Utilizing certain computational tricks, and building on earlier work with multinomial and Naive Bayes models, the authors show how the computation can be performed efficiently for tree-structured Bayesian networks.

### ACKNOWLEDGMENTS

We thank the Editor-in-Chief for the opportunity to prepare this special issue, and the staff of Hindawi for their assistance. The greatest credit is of course to the authors, who submitted contributions of the highest quality. We also thank the reviewers who have had a crucial role in the selection and editing of the ten papers appearing in the special issue.

*Jorma Rissanen*
*Peter Grünwald*
*Jukka Heikkonen*
*Petri Myllymäki*
*Teemu Roos*
*Juho Rousu*
